# A Deep Learning-Based Satellite Target Recognition Method Using Radar Data

**DOI:** 10.3390/s19092008

**Published:** 2019-04-29

**Authors:** Wang Lu, Yasheng Zhang, Can Xu, Caiyong Lin, Yurong Huo

**Affiliations:** 1 Graduate School, Space Engineering University, Beijing 101416, China; wanglu199310@163.com (W.L.); wasjrong@163.com (Y.H.); 2 Space Engineering University, Beijing 101416, China; lizhizys@139.com (Y.Z.); 13466509187@139.com (C.X.)

**Keywords:** radar automatic target recognition (RATR), high resolution range profile (HRRP), deep learning, radar data partition, gated recurrent unit (GRU)

## Abstract

A novel satellite target recognition method based on radar data partition and deep learning techniques is proposed in this paper. For the radar satellite recognition task, orbital altitude is introduced as a distinct and accessible feature to divide radar data. On this basis, we design a new distance metric for HRRPs called normalized angular distance divided by correlation coefficient (NADDCC), and a hierarchical clustering method based on this distance metric is applied to segment the radar observation angular domain. Using the above technology, the radar data partition is completed and multiple HRRP data clusters are obtained. To further mine the essential features in HRRPs, a GRU-SVM model is designed and firstly applied for radar HRRP target recognition. It consists of a multi-layer GRU neural network as a deep feature extractor and linear SVM as a classifier. By training, GRU neural network successfully extracts effective and highly distinguishable features of HRRPs, and feature visualization technology shows its advantages. Furthermore, the performance testing and comparison experiments also demonstrate that GRU neural network possesses better comprehensive performance for HRRP target recognition than LSTM neural network and conventional RNN, and the recognition performance of our method is almost better than that of other several common feature extraction methods or no data partition.

## 1. Introduction

Space target recognition is a primary function of space surveillance information systems, and satellite recognition is of critical importance on this study, especially for observation satellites. However, few open research achievements have been reported. The difficulty of this problem is that satellites are simply too small or too far away for detailed information to be recognized, and it is relatively hard to obtain effective identification data. With the development of wideband radar, not only can we locate the target position, but also get other useful radar data of targets, such as high-resolution range profile (HRRP) and inverse synthetic aperture radar (ISAR) image [1,2]. An HRRP is the phasor sum of the time returns from different scatterers on the target located within a resolution cell [3], which represents the projection of the complex returned echoes from the target scattering centers onto the range axis [4]. It contains lots of geometric structure information about the target down range, such as scatterers distribution and target size. In addition, compared with ISAR image, HRRPs have the advantages of easy acquisition, storage and processing. That’s why radar HRRP target recognition has gained high attention from radar automatic target recognition (RATR) community [5,6,7]. In summary, this paper focuses on the satellite target recognition based on radar data and proposes a novel recognition method, which mainly consists of data partition and deep learning model.

Feature extraction and selection is a basic and crucial technology for radar target recognition research. It is significant to adopt reasonable and effective features to improve recognition performance. Currently, the targets of HRRP recognition research are basically about ground or aviation targets, such as tanks and airplanes [8,9,10,11,12,13,14,15]. Satellites, as an important space target, have different motion characteristics, one of which is that their motion must follow Kepler’s law. Besides, because the fuel carried by satellites is limited, the orbital maneuver range of most satellites is restricted, which makes satellite orbits relatively stable [16,17]. Orbit information is easily accessible and non-burdensome because target range and position measurement is a basic function of radar, therefore, orbit information is a distinct and accessible feature for recognizing the satellites whose eccentricity is very small, and could be introduced into radar data partition when multiple satellites need to be recognized. In addition, radar observation pitch and azimuth angles are also important and helpful information. Utilizing radar observation angles has many benefits for target recognition, such as reducing search range and computation, relaxing attitude sensitivity and improving recognition rate [18,19]. Radar observation angular domain division have been studied in [8,11,20,21]. Uniform frame segmentation method is used in [8,11] but has been proved simple and unreasonable [22]. Angular domain division method based on statistical characteristics is studied in [20], but it would generate many matching templates and need huge computation burden and storage requirement. Correlation coefficient is firstly introduced to solve this problem in [21] and could measure similarity between HRRPs to a certain extent. However, other information may also be helpful to measure similarity and could be applied, such as angular distance. Therefore, for the data partition, the proposed method will utilize orbit information as a powerful feature, and a hierarchical clustering method with a novel distance metric, namely normalized angular distance divided by correlation coefficient (NADDCC), will be applied to segment radar angular domain.

In addition to the above available satellite and radar information, it is still necessary to further mine information in HRRP data to raise the recognition accuracy. Many studies have been done on HRRP feature extraction and selection methods. In the early days, researchers often calculated FFT-magnitude, power spectrum and a variety of high-order spectrum of HRRP data, and used them as the features of classifier for target recognition [8,9,10]. Although these engineered features could play a part in target recognition, they are dependent on researchers’ experience and techniques. Other than the features of artificial selection, machine learning algorithms have been widely utilized to extract features based on high-dimensional HRRP data [11,12,13,14,15]. Principal component analysis (PCA) is applied to extract the complex HRRPs’ feature subspace within each target-aspect sector in the literature [11]. Dictionary learning is adopted to extract the features of HRRP data, which possess high noise-robust and discrimination [12,13]. Manifold learning is employed to reduce the feature dimensions of radar HRRP [14,15]. These methods can extract appropriate features in some cases, but they are all shallow architectures that may not represent the essence of radar HRRP. Thereby, how to automatically extract the deep abstract features, which can play an important role in target recognition, has become a significant research issue.

The deep learning theory [23] advanced by Hinton could solve the abovementioned problem effectively. Deep learning allows computational models that are composed of multiple processing layers to learn representations of data with multiple levels of abstraction [24]. Because of its powerful recognition or prediction ability, deep learning methods have consistently been used in many applications, such as computer vision [25], speech recognition [26], networking and health care [27,28,29]. Some deep learning structures applied in several recent papers have been demonstrated useful for radar target recognition, such as autoencoder and its varieties [30,31], convolutional neural network (CNN) [32] and recurrent neural network (RNN) [33,34]. Due to the unique structure of RNN, it has been widely applied to process sequential data, such as action recognition [35], scene labelling [36], and language processing [37], and has achieved impressive results [38]. However, it is founded that simple conventional RNN could not learn on a wide range of dependencies, and gradients of distant time steps do not play a role in learning because of gradient vanishing. To solve this problem, long short-term memory (LSTM) and gated recurrent unit (GRU) architecture have been designed to learn long term reliance on sequential data. GRU is a variety of LSTM and retains the LSTM’s resistance to the problem of gradient vanishing. Moreover, the internal structure of GRU is simple and it requires less computation to update the hidden state, which makes the training speed faster [37,39,40]. So GRU neural network will be applied in this satellite recognition method to extract and select the deep abstract features of radar sequential HRRP data.

In this paper, a novel satellite target recognition method is proposed based on radar data. This method could make full use of radar data and apply deep learning technology to extract highly distinguishable features. Its features are summarized as follows:

(a) Satellite orbital altitude is introduced as a distinct and accessible feature for radar data partition;
(b) Radar observation angles information is fully utilized, and a hierarchical clustering method based on a new distance metric (NADDCC) is applied to segment angular domain to improve recognition rate;
(c) A novel end-to-end GRU-SVM model is designed, which uses radar HRRP data as input and target class as output, and firstly applied to identify targets based on radar HRRP data. GRU neural network is applied as a deep features extractor and support vector machine (SVM) is used as a classifier in this model. Compared with some deep neural networks such as CNN, autoencoder and denoising autoencoder, and shallow learning algorithms such as PCA [11], dictionary learning [12,13] and manifold learning [14,15], the presented model can extract the deep abstract features of HRRPs and obtain better recognition results.

The rest of this paper is organized as follows: in Section 2, the information contained in satellite orbit and radar data is analysed. In Section 3, we introduce GRU, SVM and the construction of the GRU-SVM model, and present the overall flow chart of this recognition method. In Section 4, recognition results are provided and the performance under different recognition methods and conditions is compared and analysed. In Section 5, some conclusions are drawn.

Notations: To simplify the presentation, we define the following notations used in this paper. We use bold lower case letters to represent a vector, e.g., $\mu \;\in {\;C}^{D}$, and use bold upper case letters to represent a matrix, e.g., $M\;\in {\;C}^{i\times j}$. The acronyms used in this paper are summarized in Table 1 for the sake of readability.

## 2. Analysis of Satellite and Radar Information

### 2.1. Description and Preprocessing of HRRP

**Figure 1:**
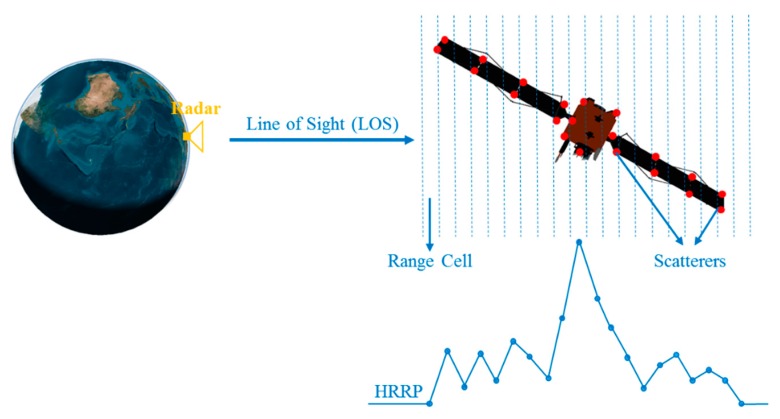
Illustration of a HRRP sample from a satellite.

HRRP is the amplitude of echo summation for target scattering centers in each range cell of wideband radar. Figure 1 shows the illustration of a HRRP sample from a satellite target. High resolution radar operates in microwave frequency band, and the size of targets or their components is much larger than the wavelength of radar. In this case, the echo characteristics of targets can be calculated by using a simplified scattering center model [3,4,9,41,42]. Therefore, for complex targets such as a satellite, the projection of an object on radar line of sight can be divided into many range cells by high resolution radar. According to the scattering center model, the scatterers in different range cells will rotate in the same way when a satellite target rotates, which causes that the echo amplitudes between range cells have certain correlation. Besides, windowing processing of the returned echoes before getting HRRP data and multiple reflections phenomena of measured HRRP data would enhance the correlation between adjacent range cells. Therefore, there is a certain temporal correlation between range cells and radar HRRP could be seen as sequential data, which is suitable to be learned by RNN-based neural networks.

The radar signatures, which return from multiple scattering centers within the same range cell, will be coherently summed as a single signature for that range cell. According to related literature [3,9], suppose the transmitted signal is $s\left(t\right)e^{{j2\pi f}_{c}t}$, the n-th complex echo in the d-th range cell (d=1, 2, ⋯, D) in the baseband can be approximated as:

(1) $${\tilde{x}}_{d}\left(t,\;n\right)\;\approx s\left(t\right)\sum_{i=1}^{L_{d}}\sigma_{\mathrm{di}}e^{-j\left\{\left(4\pi /\lambda \right)\left[R\left(n\right)+\Delta \gamma_{\mathrm{di}}\left(n\right)\right]\right\}}$$

where s(t) is the complex envelop which could be approximated as unchanged for all scatterers in one range cell. λ represents the wavelength of wideband radar and $f_{c}$ is the carrier frequency of radar signal. $L_{d}$ denotes the number of scatterers in the d-th range cell. $\sigma_{\mathrm{di}}$ represents the intensity of the i-th scatterer in the d−th range cell. R(n) is the radial distance between the radar and target reference center in the n-th echo. ${\Delta \gamma }_{\mathrm{di}}\left(n\right)$ is the radial displacement of the i-th scatterer of the d-th range cell in the n-th echo. Usually, s(t) is a rectangular pulse signal with unit intensity and could be omitted. After eliminating the initial phase of the n-th echo $e^{-j\left(4\pi /\lambda \right)R\left(n\right)}$, the n-th HRRP can be defined as:

(2) $$\begin{array}{cc} x\left(n\right) & =[x_{1}\left(n\right){,x}_{2}\left(n\right),\cdots {,x}_{D}\left(n\right)]\\ & =\left[\left|\sum_{i=1}^{L_{1}}\sigma_{1i}e^{j\phi_{1i}\left(n\right)}\right|,\left|\sum_{i=1}^{L_{2}}\sigma_{2i}e^{j\phi_{2i}\left(n\right)}\right|,\cdots ,\left|\sum_{i=1}^{L_{D}}\sigma_{\mathrm{Di}}e^{j\phi_{\mathrm{Di}}\left(n\right)}\right|\right] \end{array}$$

Several sensitivity issues of HRRP should be focused on when the HRRP target recognition task is carried out. The first one is time-shift sensitivity. To decrease the computation complexity, HRRP is only a portion of received radar echo extracted by a range window, which contains the target signal. Thus the position of the target signal in HRRP would change with the measurement. However, it would be better for feature learning that all the training samples meet a uniform parameter model. So we adopt envelope alignment method [43] as time-shift compensation technique in this paper, which is achieved based on the summation average of multiple HRRPs cross-correlations. The second one is amplitude-scale sensitivity. It is caused by the fact that many factors could influence the intensity of an HRRP, such as target distance, radar transmitting power, radar antenna gain, radar receiver gain and radar system losses. It makes that HRRPs measured by different radars or under different conditions would have different amplitude-scales. In order to deal with amplitude scale sensitivity, each HRRP is normalized by energy normalization method [3,9]. Suppose an HRRP is defined as $x\left(n\right)\;=\;\left[x_{1}\left(n\right){,x}_{2}\left(n\right),\cdots {,x}_{D}\left(n\right)\right]$, then its energy normalization preprocessing result $\tilde{x}\left(n\right)$ is shown as follows:

(3) $$\begin{array}{cc} \tilde{x}\left(n\right) & =\left[{\tilde{x}}_{1}\left(n\right),{\tilde{\;x}}_{2}\left(n\right),\;\cdots \;,{\tilde{\;x}}_{D}\left(n\right)\right]\\ & =\left[\frac{x_{1}\left(n\right)}{\sqrt{\sum_{i=1}^{D}x_{i}{\left(n\right)}^{2}}},\;\frac{x_{2}\left(n\right)}{\sqrt{\sum_{i=1}^{D}x_{i}{\left(n\right)}^{2}}},\;\cdots \;,\;\frac{x_{D}\left(n\right)}{\sqrt{\sum_{i=1}^{D}x_{i}{\left(n\right)}^{2}}}\right] \end{array}$$

**Figure 2:**
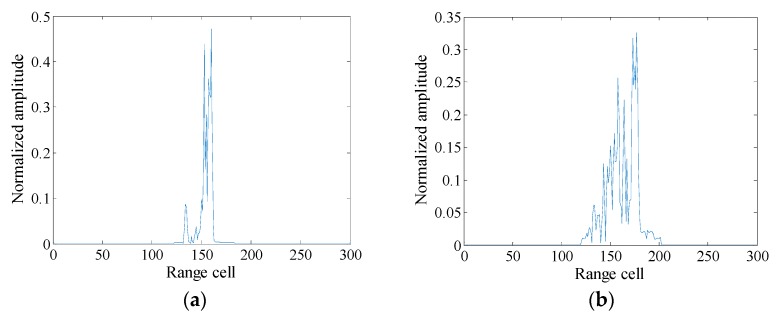
HRRP sample examples of satellites. (**a**) HRRP sample example of satellite No.1; (**b**) HRRP sample example of satellite No.2.

After the above preprocessing step, the HRRP sample examples of satellites are shown in Figure 2. The last and toughest one is called target-attitude sensitivity. This issue will be analyzed in detail in Section 2.3 and further alleviated.

### 2.2. Analysis and Statistics of Satellite Orbit

**Figure 3:**
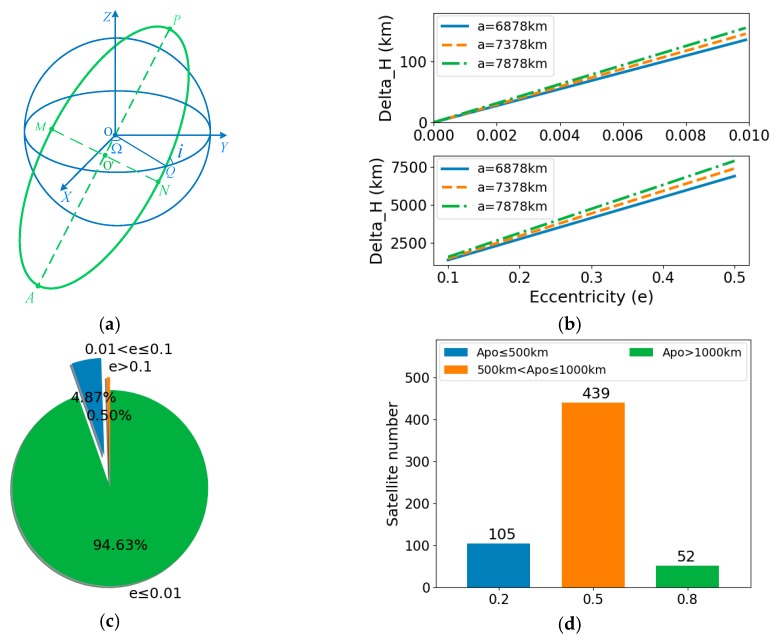
Analysis and statistical results of observation satellite orbit. (**a**) Satellite orbital parameters description; (**b**) Variation of altitude difference with eccentricity when semi-major axis is given; (**c**) Eccentricity statistical results; (**d**) Apogee altitude statistical results.

Currently, thousands of artificial satellites move around the Earth for the purposes of communication and navigation, information relay, missile warning, on-orbit service and so on. Among them, observation satellites are the key targets, which need to be identified for space surveillance information systems. Unlike ground or aviation targets, satellites’ motion must follow Kepler’s law, that is, they basically move along certain orbit. As shown in Figure 3a, satellite orbit is mainly described with the following parameters:

(1) Semi-major axis (a): the distance from the apogee or perigee to orbital center which describes the size of satellite orbit;
(2) Eccentricity (e): describes the shape of satellite orbit;
(3) Inclination (i): the angle between the orbital plane and the equatorial plane which determines the accessible area of a satellite;
(4) Longitude of ascending node (Ω): the right ascension at the intersection of the orbital plane and the equatorial plane.
(5) Argument of perigee (ω): angular distance of orbit perigee and ascending node.

Based on the above parameters, the altitude range of a satellite can be computed by:

(4) $$\{\begin{array}{c} H_{\max}\;=\;a\cdot \left(1\;+\;e\right)\;{-\;R}_{e}\\ H_{\min}\;=\;a\cdot \left(1\;-\;e\right)\;{-\;R}_{e} \end{array}$$

where $H_{\max}$ and $H_{\min}$ respectively refer to the maximum and minimum altitude of a satellite. $R_{e}$ denotes the radius of Earth. In this way, the variation of satellite altitude difference with eccentricity can be calculated when the value of semi-major axis is given, as shown in Figure 3b. It can be seen that only when eccentricity is small enough can the orbital altitude information be used for identification, so it is still necessary to investigate the eccentricity distribution of current observation satellites. UCS Satellite Database [44] has made detailed statistics of satellites currently orbiting Earth. Eccentricity and apogee altitude statistical results of observation satellites are shown in Figure 3c,d. It shows that the eccentricity of most satellites is lower than 0.01, especially for optical or radar imaging satellites. Therefore, orbital altitude information is an available and useful feature for recognizing observation satellites.

**Figure 4:**
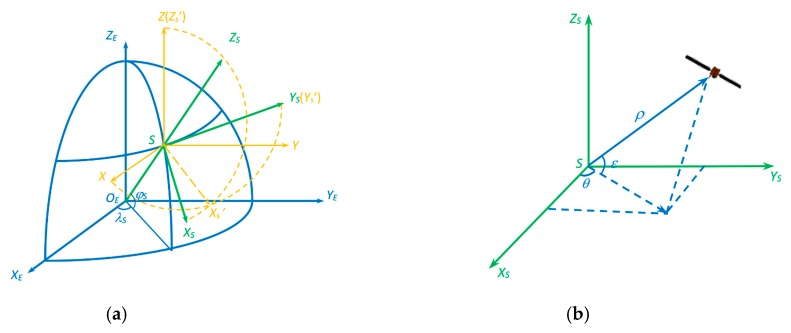
Coordinate transformation diagrams. (**a**) Radar body coordinate system transforms into geocentric coordinate system; (**b**) Radar polar coordinate transforms into radar body Cartesian coordinate system.

For non-cooperative satellite targets, it is difficult to compute orbital altitude by orbital parameters because orbital parameters are not necessarily known or constant. However, it may become easy when radar is applied. For radar, distance measurement is a basic function and its observation angles are known. Satellite orbital altitude could be obtained by multiple coordinate transformation based on radar measurement data and the main coordinate transformation diagrams are shown in Figure 4. The detailed orbital altitude calculation process is shown in Appendix A.

### 2.3. Target-Attitude Sensitivity and Radar Observation Angular Domain Segmentation

Target-attitude sensitivity is one of the most difficult problems in radar HRRP target recognition research. According to the scattering center model [3,4,9,41,42], the variation of target attitude will lead to different range shifts for different scattering centers on the target, even within the attitude region where the scattering center structure remains unchanged (that is, without migration throuth resolution cells, MTRC). Specifically, for the HRRP of the m−th returned echo, suppose it is $x\left(m\right)\;=\;\left[x_{1}\left(m\right){,x}_{2}\left(m\right),\cdots {,x}_{D}\left(m\right)\right]$, then the echo power of the n-th range cell could be computed by:

(5) $${\left|x_{n}\left(m\right)\right|}^{2}{\;=\;x}_{n}\left(m\right)x_{n}^{*}\left(m\right)\;=\;\sum_{i=1}^{L_{n}}\sigma_{\mathrm{ni}}^{2}\;+\;2\sum_{i=2}^{L_{n}}\sum_{k=1}^{i-1}\sigma_{\mathrm{ni}}\sigma_{\mathrm{nk}}\cos\left[\theta_{\mathrm{nik}}\left(m\right)\right]$$

where * represents complex conjugate operation. $\sigma_{\mathrm{ni}}$ denotes the intensity of the i-th scatterer in the n-th range cell. $\overset{L_{n}}{\underset{i=1}{\sum }}\sigma_{\mathrm{ni}}^{2}$ is the conjugate product of the sub-echo for all scatterers in the n-th range cell, which represents the intensity sum of each scatterer and is relatively stable. However, $\cos\left[\theta_{\mathrm{nik}}\left(m\right)\right]$ of the second item, which is called cross-term, will change with the variation of m, where $\theta_{\mathrm{nik}}\left(m\right)$ represents the phase difference of the i-th scatterer and the k-th scatterer of the n-th range cell in the m-th returned echo. Therefore, an HRRP, which is the amplitude of coherent sum of the complex returned echoes from scatterers in a range cell, can be changed substantially.

The target-attitude sensitivity problem makes it hard to recognize satellite targets base on HRRP data and needs to be focused on. It has been founded that average range profile [22] is helpful to improve attitude stability of HRRP, because the sum of the irrelevant echo power of the cross-terms will greatly weaken the effect of cross-items. As mentioned earlier, the correlation coefficient between average range profiles can be used to measure similarity of HRRPs [21]. For a set of HRRP samples {x(0), x(1), ⋯, x(M−1)}, which are translationally aligned and without MTRC, we represent them as $x\left(i\right)\;=\;\left[x_{1}\left(i\right){,x}_{2}\left(i\right),\cdots {,x}_{D}\left(i\right)\right],\;i\;=\;0,1,\cdots ,M-1$, then its average range profile is [22] defined as:

(6) $$\mu \;=\;\left[\sqrt{\frac{1}{M}\overset{M-1}{\underset{m=0}{\sum }}{\left|x_{1}\left(m\right)\right|}^{2}},\;\sqrt{\frac{1}{M}\overset{M-1}{\underset{m=0}{\sum }}{\left|x_{2}\left(m\right)\right|}^{2}},\;\cdots ,\;\sqrt{\frac{1}{M}\overset{M-1}{\underset{m=0}{\sum }}{\left|x_{D}\left(m\right)\right|}^{2}}\right]$$

Research also suggests that utilizing radar observation angles information is beneficial, such as reducing search range and computation, relaxing target-attitude sensitivity and improving recognition rate [18,19].

Through the above analysis, for the target-attitude sensitivity problem of HRRP, we propose a hierarchical clustering method with a novel distance metric, namely the normalized angular distance divided by correlation coefficient (NADDCC), to segment radar observation angular domain. This distance metric includes both angular distance information and correlation coefficient between HRRP average range profiles, which could measure similarity between HRRP average range profiles better. For average range profiles $\mu_{i}=\left[\mu_{i}\left(1\right){,\;\mu }_{i}\left(2\right),\;\cdots {,\;\mu }_{i}\left(D\right)\right]$ whose observation azimuth and elevation angles are $\theta_{i}$ and $\varepsilon_{i}$, and $\mu_{j}=\left[\mu_{j}\left(1\right){,\;\mu }_{j}\left(2\right),\;\cdots {,\;\mu }_{j}\left(D\right)\right]$ whose observation azimuth and elevation angles are $\theta_{j}$ and $\varepsilon_{j}$, their angular distance $d_{\mathrm{angle}}\left(\mu_{i}{,\;\mu }_{j}\;\right)$ and correlation coefficient $\rho \left(\mu_{i}{,\;\mu }_{j}\;\right)$ are defined as follows:

(7) $$d_{\mathrm{angle}}\left(\mu_{i}{,\;\mu }_{j}\;\right)\;=\;\sqrt{{\left(\theta_{i}{-\theta }_{j}\right)}^{2}\;+\;{\left(\varepsilon_{i}{-\varepsilon }_{j}\right)}^{2}}$$

(8) $$\rho \left(\mu_{i}{,\;\mu }_{j}\;\right)\;=\;\frac{\mu_{i}\mu_{j}^{T}}{∥\mu_{i}∥{}_{2}∥\mu_{j}∥{}_{2}}\;=\;\frac{\sum_{n=1}^{D}\left[\mu_{i}\left(n\right)\mu_{j}\left(n\right)\right]}{∥\mu_{i}∥{}_{2}∥\mu_{j}∥{}_{2}}$$

The larger the correlation coefficient and the smaller the angular distance, the higher the similarity of HRRP average range profiles. In addition, the distance metric applied for hierarchical clustering usually need to satisfy some necessary properties, such as non-negativity, identity and symmetry. In order to make the two distances work equally, they are normalized by means of dividing by their respective maximum. Taking into account the above considerations, the distance metric presented in this paper is designed as follows:

(9) $$d\left(\mu_{i}{,\mu }_{j}\right)=\frac{d_{\mathrm{angle}}\left(\mu_{i}{,\;\mu }_{j}\;\right)/\max\left(d_{\mathrm{angle}}\right)}{\rho \left(\mu_{i}{,\;\mu }_{j}\;\right)/\max\left(\rho \right)}=\frac{\sqrt{{\left(\theta_{i}{-\theta }_{j}\right)}^{2}+{\left(\varepsilon_{i}{-\varepsilon }_{j}\right)}^{2}}\cdot ∥\mu_{i}∥{}_{2}∥\mu_{j}∥{}_{2}\cdot \max\left(\rho \right)}{\sum_{n=1}^{D}\left[\mu_{i}\left(n\right)\mu_{j}\left(n\right)\right]\cdot \max\left(d_{\mathrm{angle}}\right)}$$

Hierarchical clustering algorithm adopts bottom-up aggregation strategy. Firstly, each HRRP average range profile is regarded as an initial cluster, and then two nearest clusters are found and merged at each step of algorithm operation. The process is repeated until the number of preset clusters is reached. The clustering process can be summarized as follows:
**Hierarchical Clustering Algorithm****Input:** HRRP average range profile sample set $S\;=\;\left\{\mu_{1}{,\;\mu }_{2},\;\cdots {,\;\mu }_{m}\right\}$;           
distance metric $d_{\mathrm{avg}}\left(C_{i}{,\;C}_{j}\right)\;=\;\frac{1}{\left|C_{i}\right|\left|C_{j}\right|}\underset{\mu_{i}\in C_{i}}{\sum }\underset{\mu_{j}\in C_{j}}{\sum }d\left(\mu_{i}{,\;\mu }_{j}\right)$;           
cluster number k.**Process: for**j = 1, 2, ⋯, m**do**
                    
$C_{j}\;=\;\left\{\mu_{j}\right\}$
               
**end for**
               
**for**
i = 1, 2, ⋯, m
**do**                    
**for**
j = i+1, ⋯, m
**do**                         
$M\left(i,\;j\right){\;=\;d}_{\mathrm{avg}}\left(C_{i}{,\;C}_{j}\right)$;                         
M(j, i) = M(i, j)                    
**end for**               
**end for**               
set the number of current clusters: q=m               
**while**
q > k
**do**                    
find the two nearest cluster $C_{i*}$ and $C_{j*}$;                    
merge $C_{i*}$ and $C_{j*}$: $C_{i*\;}{=\;C}_{i*}\;\cup {\;C}_{j*}$;                    
**for**
${j\;=\;j}^{*}{+1,\;j}^{*}+2,\;\cdots ,\;q$
**do**                         
renumber cluster $C_{j}$ as $C_{j-1}$                    
**end for**                    
delete line $j^{*}$ and column $j^{*}\;$ of matrix M                    
**for**
j = i+1, ⋯, q-1
**do**                         
$M\left(i^{*},\;j\right){\;=\;d}_{\mathrm{avg}}\left(C_{i*}{,\;C}_{j}\right)$;                         
$M\left({j,\;i}^{*}\right)\;=\;M\left(i^{*},\;j\right)$                    
**end for**                    
q = q − 1                
end while**Output:** clusters $C\;=\;\left\{C_{1}{,\;C}_{2},\;\cdots {,\;C}_{k}\right\}$


## 3. GRU-SVM Model

The designed GRU-SVM model is a combination of a GRU neural network as a deep feature extractor and SVM as a classifier. It makes the best of the advantages of the GRU deep neural network and SVM to extract the deep abstract features and complete an accurate classification. This model is described in detail below.

### 3.1. GRU

**Figure 5:**
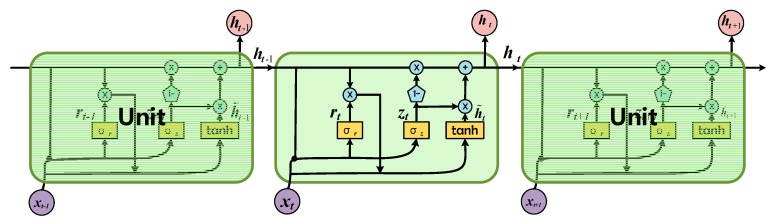
Gated recurrent unit.

As mentioned above, GRU is designed in [37,45] to learn long term reliance on sequential data, and its overall performance is better than LSTM and simple conventional RNN [39,40]. Figure 5 shows a GRU model. There are only two gates in a GRU, namely the update gate z and the reset gate r. The update gate is utilized to modulate the previous information inside the unit. The larger the value of update gate, the more the status information of the previous moment insides. The reset door is used to control how much previous state information will be forgotten. The smaller the value of the reset gate, the more the previous state information is forgotten.

The update gaze $z_{t}$ and reset gate $r_{t}$ at the time t are defined as:

(10) $$\{\begin{array}{c} z_{t}\;=\;\sigma \left(W_{z}x_{t}{\;+\;U}_{z}h_{t-1}\right)\\ r_{t}\;=\;\sigma \left(W_{r}x_{t}{\;+\;U}_{r}h_{t-1}\right) \end{array}$$

where W and U are weight matrices. x denotes input data. The hidden state $h_{t}$ and candidate hidden state ${\tilde{h}}_{t}$ in GRU are calculated respectively as follows:

(11) $$\{\begin{array}{l} h_{t}\;=\;\left(1\;-\;z\right)h_{t-1}{\;+\;z}_{t}{\tilde{h}}_{t}\\ {\tilde{h}}_{t}\;=\;\tan h\left(W_{h}x_{t}{\;+\;U}_{t}\left(r_{t}{\;*\;h}_{t-1}\right)\right) \end{array}$$

where * represents element-wise product. The σ(·) and tanh(·) are two different activation functions which can be defined as:

(12) $$\begin{array}{l} \sigma \left(x\right)\;=\;\frac{1}{{1\;+\;e}^{x}}\\ \tan h\left(x\right)\;=\;\frac{{1\;-\;e}^{2x}}{{1\;+\;e}^{2x}} \end{array}$$

In this paper, GRU is employed in the GRU neural network to extract effective features based on HRRP sequential data.

### 3.2. SVM

The support vector machine (SVM) was developed by Vapnik [46] for binary classification. Its objective is to find the optimal hyper-plane f(w,x) = w · x + b to separate two classes in a given dataset, where x is the feature vector. SVM learns the parameters w and b by solving the following constrained optimization problem:

(13) $$\begin{array}{c} \min\frac{1}{p}w^{T}w\;+\;C\sum_{i=1}^{p}\xi_{i}.\\ s.t\;\begin{array}{l} y_{i}^{\prime }\left(w\;\cdot \;x\;+\;b\right)\;\geq {\;1\;-\;\xi }_{i}\\ \xi_{i}\;\geq \;0,\;i\;=\;1,\cdots ,\;p \end{array} \end{array}$$

where $w^{T}w$ is the Manhattan norm, C is the penalty parameter, and  ξ is the cost function. The corresponding unconstrained optimization problem of Equation (18) is as follows:

(14) $$\min\frac{1}{p}w^{T}w\;+\;C\overset{p}{\underset{i=1}{\sum }}\max\left({0,\;1\;-\;y}_{i}^{\prime }\left(w^{T}{\;x}_{i}\;+\;b\right)\right)$$

where $y^{\prime }$ is the actual label, and $w^{T}\;x\;+\;b$ is the predictor function. This equation is known as L1-SVM, with the standard hinge loss. Its differential counterpart L2-SVM is given by the following equation:

(15) $$\min\frac{1}{p}{∥w∥}_{2}^{2}\;+\;C\overset{p}{\underset{i=1}{\sum }}\max{\left({0,\;1\;-\;y}_{i}^{\prime }\left(w^{T}{\;x}_{i}\;+\;b\right)\right)}^{2}$$

where ${∥w∥}_{2}$ is the Euclidean norm (also known as L2 norm), with the squared hinge loss.

Despite being intended for binary classification, SVM may be used for multi-classification as well. One approach to achieve this is the use of kernel tricks, which convert a linear model into a non-linear model by applying kernel functions. However, we just use LSVM instead of utilizing kernel tricks in this paper, because LSVM does not employ any feature extraction and transformation and can serve as a simple baseline for evaluating the quality of extracted features. A one-vs-one scheme is employed to achieve multi-classification in this paper, which establishes the binomial classifier for every two classification.

### 3.3. GRU-SVM Model Construction

**Figure 6:**
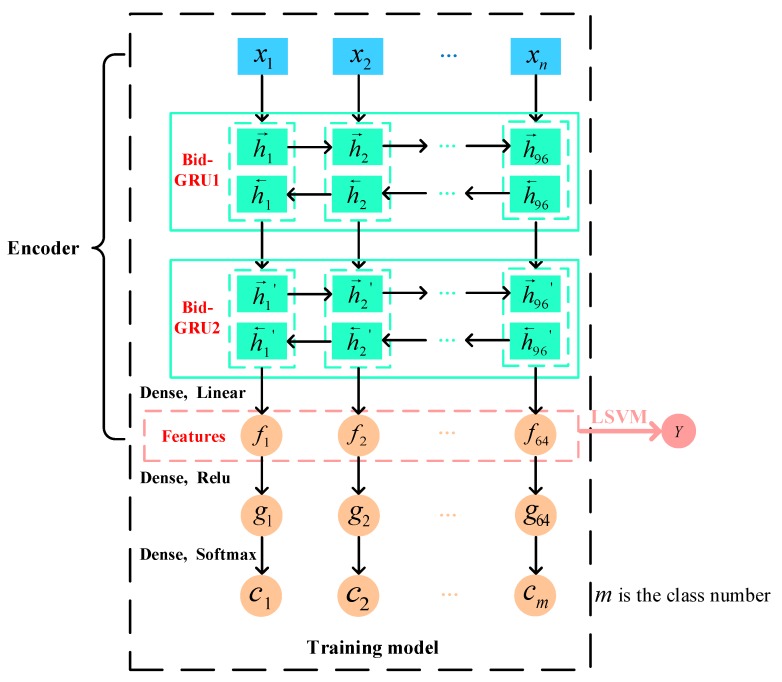
The structure of GRU-SVM model.

In this paper, a novel end-to-end GRU-SVM model has been designed and firstly applied to recognize targets based on radar HRRP data. The structure of GRU-SVM model is shown in Figure 6. The composition of this model includes two parts: GRU neural network as a feature extractor and LSVM as a classifier. GRU neural network is constituted of input layer, four hidden layers and output layer, where GRU hidden layer and fully connected layer (dense layer) are included. And the input layer, two GRU hidden layers and a fully connected layer make up the encoder module, whose output are defined as the features extracted by GRU neural network. In order to make the training model more accurate, we apply the bidirectional scheme demonstrated in reference [47] in GRU hidden layers (see Bid-GRU layer in Figure 6). In addition, two fully connected layers have been employed after the encoder to extract good features. And the last layer, also called output layer, would output the satellite classifications by adopting softmax activation function. That is, GRU neural network is trained in a supervised way. The reasons why we choose to apply the softmax classifier to train GRU neural network are that it’s an excellent multi-class classifier and its common loss function, namely categorical cross-entropy loss, is more sensitive to classification output than the hinge loss of LSVM, which means that it is always optimizing the network parameters to reduce loss during training. Therefore, the utilization of softmax classifier may make the features extracted by the encoder more highly distinguishable. By training GRU neural network, the encoder will produce the length-fixed feature vectors which contain sufficient information for target recognition. LSVM classifier takes feature vectors as input and produce classification results. It mainly has two roles: one the one hand, LSVM classifier could have outstanding generalization performance for the testing data after it is trained with the extracted features and their corresponding labels; on the other hand, LSVM classifier is applied as a simple baseline for evaluating the quality of features extracted by different methods here, because it does not employ any feature extraction and transformation. However, the softmax classifier in this GRU neural network may be not suitable for comparing the quality of extracted features on account of the existence of nonlinear activation function. Linear φ(·), relu ϕ(·) and softmax ψ(·) activation functions are employed in this model and defined as follows:

(16) $$\begin{array}{l} \varphi \left(x\right)\;=\;\underset{i}{\sum }x_{i}w_{i}\;+\;b\\ \phi \left(x\right)\;=\;\max\left(0,\;x\right)\\ \psi {\left(x\right)}_{j}\;=\;\frac{e^{x_{j}}}{\sum_{k=1}^{K}e^{x_{k}}} \end{array}$$

**Figure 7:**
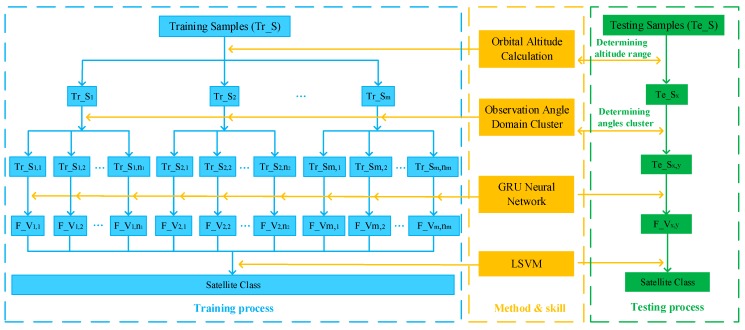
Overall framework of this satellite target recognition method. Note: Tr_S, Te_S and F_V represent the training samples, testing sample and feather vector, respectively. Their subscripts denote different data partition results, where the first subscript represents the partition results of orbital altitude and the second represents the partition results of hierarchical clustering.

After the analysis and description of Section 2 and Section 3, the overall framework of this proposed satellite target recognition method is shown in Figure 7. It can be divided into three parts, namely training process, testing process and the methods and techniques used therein. Orbital altitude calculation and observation angular domain segmentation are applied to divide HRRP data, including training samples and testing samples. The detailed orbital altitude calculation process is shown in the Appendix A. On this foundation, GRU neural network is trained to get the deep abstract features based on divided HRRP training data. Then, the classification results of testing data could be obtained by the trained GRU neural network and LSVM.

## 4. Experimental Results and Discussion

In this section, test experiments will be carried out to obtain the performance of the proposed recognition method. After dividing training data and completing the training process of GRU-SVM model, the recognition accuracy of testing data set will be gotten according to the testing process in Figure 7. Furthermore, performance testing and comparison experiments of different conditions and recognition methods also have been done to better illustrate the advantages of this recognition method.

### 4.1. Data Generation and Partition

**Figure 8:**
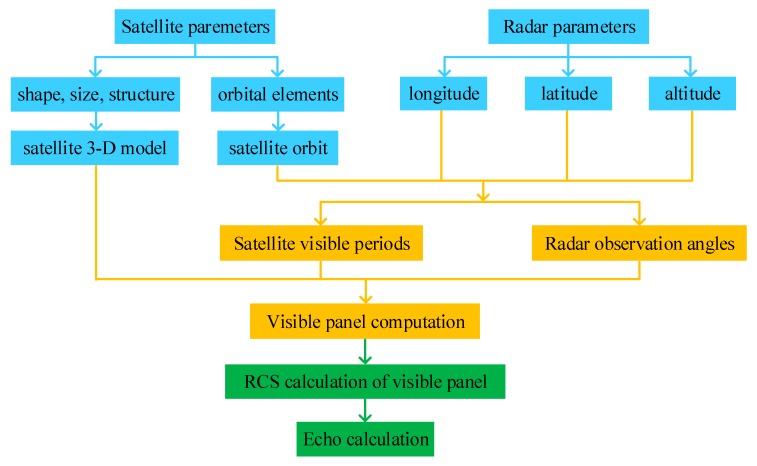
Flow chart of data generation.

**Figure 9:**
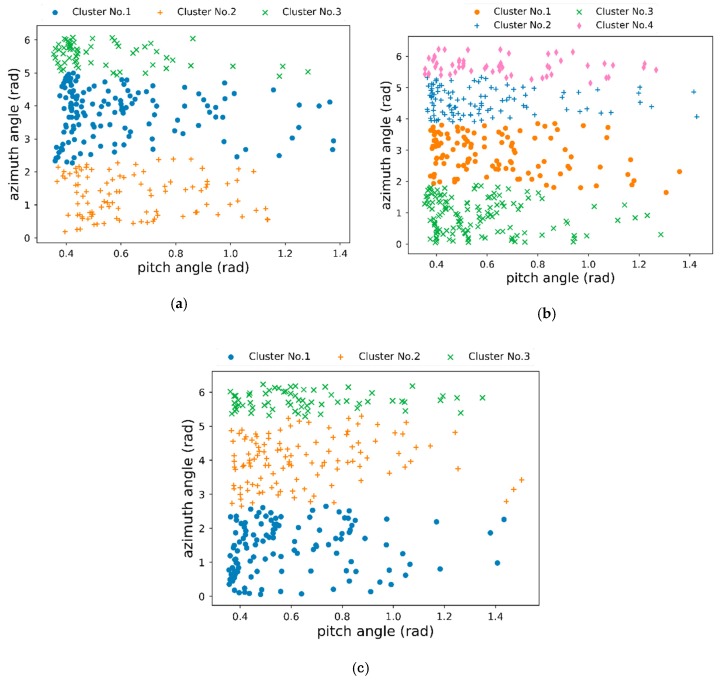
Partition results of training radar data. (**a**)${\;H}_{\mathrm{Sat}}\leq 500\mathrm{km}$; (**b**)${\;500<\;H}_{\mathrm{Sat}}\leq 1000\mathrm{km}$; (**c**)${\;H}_{\mathrm{Sat}}>1000\mathrm{km}$.

Considering the difficulty of obtaining satellite HRRP measured data, we utilize reliable simulation radar HRRP data from ten satellites that simulated by an X-band radar with a center frequency of 10 GHZ and a bandwidth of 1 GHz. The main parameters of radar and these satellites are listed in Table 2 and the detailed flow chart of radar data generation is shown in Figure 8. The observational relationship between satellite and radar is calculated based on their parameters. Then, radar cross section (RCS) and echo are computed and radar data is obtained. In our experiments, each satellite target has 70,000 HRRP samples and each HRPP is a 300-dimensional vector. The data preprocessing operation has been done as described in Section 2.1. 80% of radar data will be applied as training set and others as testing set. For training set, data partition would be done by orbital altitude calculation and observation angular domain segmentation. Figure 9 shows the data partition results. The orbital altitude of this ten satellites is divided into three ranges in this paper, namely $H_{\mathrm{Sat}}\leq 500\mathrm{km}$, $500<H_{\mathrm{Sat}}\leq 1000\mathrm{km}$ and $H_{\mathrm{Sat}}>1000\mathrm{km}$. Meanwhile, a hierarchical clustering method with a novel NADDCC distance metric has been implemented for HRRP data in each orbital altitude range to get clustering datasets. It should be noted that a set of radar observation angles is shared by continuous 700 radar HRRP data. Thus, we could be decide which cluster the test data belongs to when determining its altitude range and angles cluster.

### 4.2. Training Assessment of GRU Neural Network

After data partition, GRU neural network could be trained with these clustering datasets as input. In order to train the network faster and more accurately, we apply the following deep neural network training techniques in this paper:

(1) 20% of training data is used as validation set to adjust the hyper-parameters;
(2) Drop-out is employed for the two GRU layers to avoid the problem of overfitting and set to 0.25;
(3) Batch normalization is inserted after each layer to accelerate the training.

**Figure 10:**
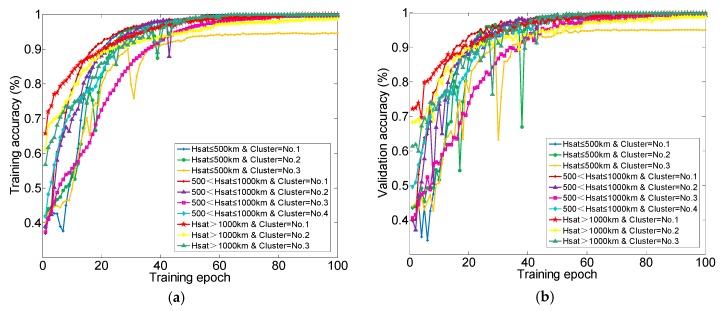

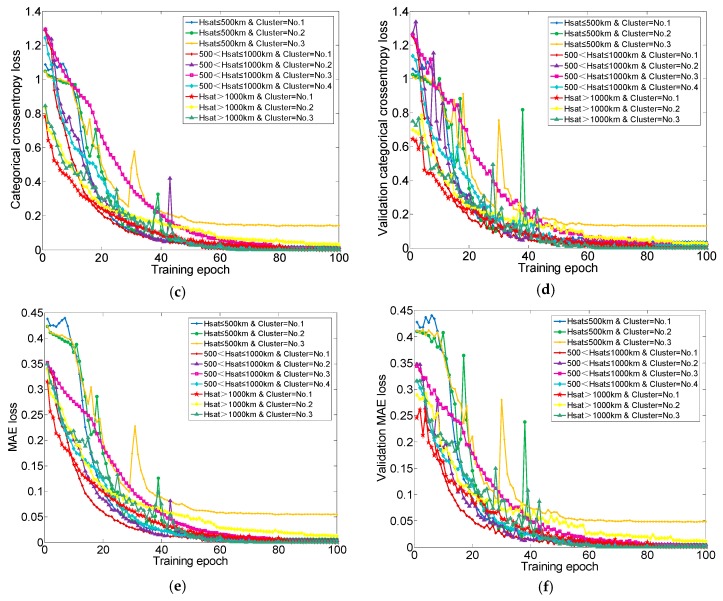
Training records of 100 epochs based on different training dataset. (**a**) Training accuracy; (**b**) Validation accuracy; (**c**) Categorical crossentropy loss of training set; (**d**) Categorical crossentropy loss of validation set; (**e**) MAE loss of training dataset; (**f**) MAE loss of validation set.

For multi-classification problems, recognition accuracy, categorical cross-entropy loss ${\mathrm{Loss}}_{\mathrm{CC}}$ and mean absolute error (MAE) loss ${\mathrm{Loss}}_{\mathrm{MAE}}$ are often applied to assess classification result. They are defined as follows:

(17) $$\begin{array}{l} \mathrm{accuracy}\;=\;N/M\\ {\mathrm{Loss}}_{\mathrm{CC}}\;=\;-\overset{M}{\underset{i=1}{\sum }}\overset{m}{\underset{j=1}{\sum }}y_{\mathrm{ij}}{\log\hat{y}}_{\mathrm{ij}}\\ {\mathrm{Loss}}_{\mathrm{MAE}}\;=\;\frac{1}{M}\overset{M}{\underset{i=1}{\sum }}\left|y_{i}{-\hat{y}}_{i}\right| \end{array}$$

where M is the total number of current training samples and N is the number of the samples predicted correctly. ${\hat{y}}_{i}$ denotes the prediction value of the ith sample and $y_{i}$ denotes expected value. m represents the class number and is usually greater than or equal to 3. In order to reduce the loss and improve accuracy in the training process, it is necessary to choose a suitable optimizer for GRU neural network. Adam is an excellent optimizer which combines the main advantages of the previous deep learning optimizers AdaGard and RMSProp. Thus, the Adam optimizer is used with the initial learning rate ${1\times 10}^{-3}$. When evaluation indicator is not improving, the learning rate will decrease in multiple. The training records of 100 epochs are shown in Figure 10. It could be found that the training and validation accuracy raises with the increase of training epoch and converges to a high accuracy value. Correspondingly, the categorical cross-entropy loss and MAE of training and validation data decrease with the increase of training epoch. These results all confirm that GRU neural network is well trained for all training clustering datasets.

### 4.3. Recognition Results and Comparative Analysis

The performance of this recognition method could be obtained on the basis of data partition and GRU neural network training, and a full comparative analysis of the recognition results under different conditions and recognition methods is also made in this section.

#### 4.3.1. Classification Results of this Recognition Method

**Figure 11:**
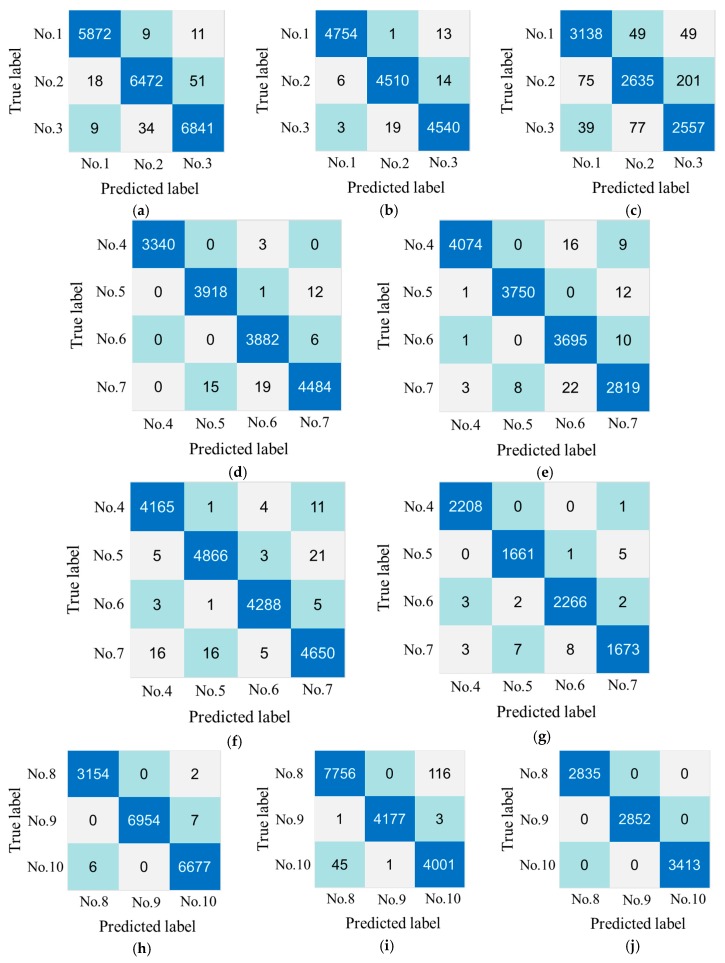
Confusion matrices of untrained data tested by the following training model. $H_{\mathrm{Sat}}\leq 500\mathrm{km}$: (**a**) Cluster=No.1; (**b**) Cluster=No.2; (**c**) Cluster=No.3. ${500\text{<}H}_{\mathrm{Sat}}\leq 1000\mathrm{km}$:(**d**) Cluster=No.1; (**e**) Cluster=No.2; (**f**) Cluster=No.3; (**g**) Cluster=No.4. $H_{\mathrm{Sat}}>1000\mathrm{km}$: (**h**) Cluster=No.1; (**i**) Cluster=No.2; (**j**) Cluster=No.3.

After completing radar data partition and GRU neural network training, a series of trained neural network models can be obtained based on training datasets of different clusters. According to the overall recognition framework in Figure 7, the orbital altitude calculation and observation angles assignment of testing data will be carried out, which is for determining which training model to extract their deep abstract features. Then, classification testing would be implemented by LSVM based on these features and corresponding recognition results of testing data could be gotten. The confusion matrices of these data tested by all training models are shown in Figure 11 and the corresponding recognition accuracy are listed in Table 3. It can be seen that testing data could be correctly divided into its corresponding trained model by orbital altitude calculation and observation angles assignment. Meanwhile, these ten satellites can be well recognized and achieve a total accuracy of 99.2%.

#### 4.3.2. Comparative Analysis of Classification Results

For the feature extraction based on radar HRRP data of satellites, it is unclear whether the GRU neural network performs better than the conventional RNN or LSTM neural networks or not. Therefore, we still need to conduct performance testing experiments for these three RNN-based neural networks. In this paper, we focus on the quality of features extracted by them and the training time they need, where feature quality could be reflected by the recognition accuracy after LSVM classifier. Radar HRRP data under different partition conditions are applied as the input of these neural networks. To better compare their training complexity, they are trained with the same training data scale, computing resources and network training parameters when the same radar HRRP data are used as input. The performance testing results are shown in Table 4. 

These results demonstrate that the features extracted by GRU neural network are the most distinguishable, and thus GRU-SVM model achieve the maximum recognition accuracy. Although conventional RNN needs the least training time, it cannot learn a good presentation of the satellite HRRP sequential data because of the network structure limitation and vanishing gradient. The performance in satellite HRRP feature extraction of the LSTM neural network is inferior to that of GRU neural network, and LSTM needs the maximum training time owing to its complex network structure. Therefore, considering the above comparison results, we choose GRU neural network as the feature extractor of satellite HRRP sequential data and just need to compare the performance of GRU neural network and other common feature extraction methods later.

**Figure 12:**
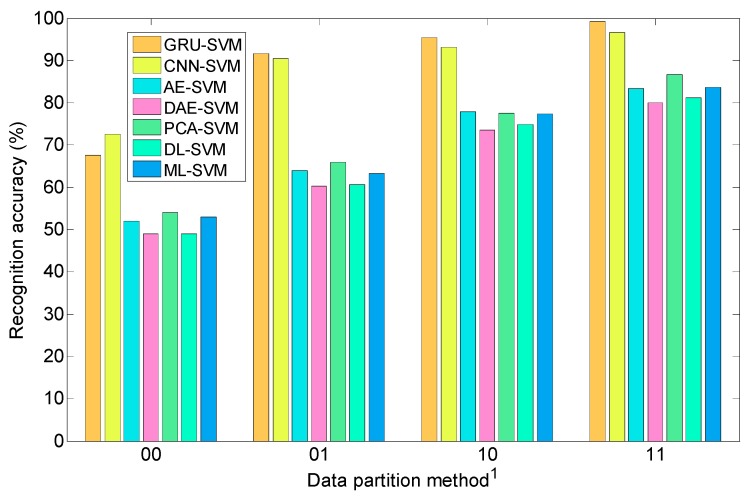
Statistical recognition result of seven methods under different conditions. ^1^ The first number represents whether to use orbital altitude calculation and the second represents whether to utilize observation angles clustering, for example ‘10′ denotes orbital altitude calculation has been applied but clustering is not applied.

In order to demonstrate the effect and advantages of this proposed recognition method, recognition performance for testing data is compared when different methods are applied to recongnize satellites under different conditions. Four deep neural networks, namely GRU neural network, CNN, autoencoder (AE) and denoising autoencoder (DAE), and three shallow models, including PCA, dictionary learning (DL) and manifold learning (ML), serve as feature extractor and LSVM is used to classify these satellites based on these extracted features. To ensure fairness in performance comparison, the number of some layers in these neural networks should be as same as possible, such as GRU hidden layer, CNN layer and encoder/decoder layer; These seven feature extraction methods could all reduce the 300-dimensional HRRP samples to same dimension, for example 64 dimensions in this paper. Table 5 shows the detailed recognition accuracy of these seven methods under various conditions and the corresponding statistical comparable results are shown in Figure 12. We can make the following conclusions from these two charts:

(1) Orbital altitude calculation and observation angles clustering technology are favourable for improving the recognition rate of satellites for all seven methods, which verifies the validity of radar data partition;
(2) Compared with the latter six methods, GRU-SVM model has good recognition performance for these ten satellites. Therefore, its total recognition accuracy rate is almost highest among these seven methods no matter whether orbital altitude calculation or observation angles clustering is applied.

**Figure 13:**
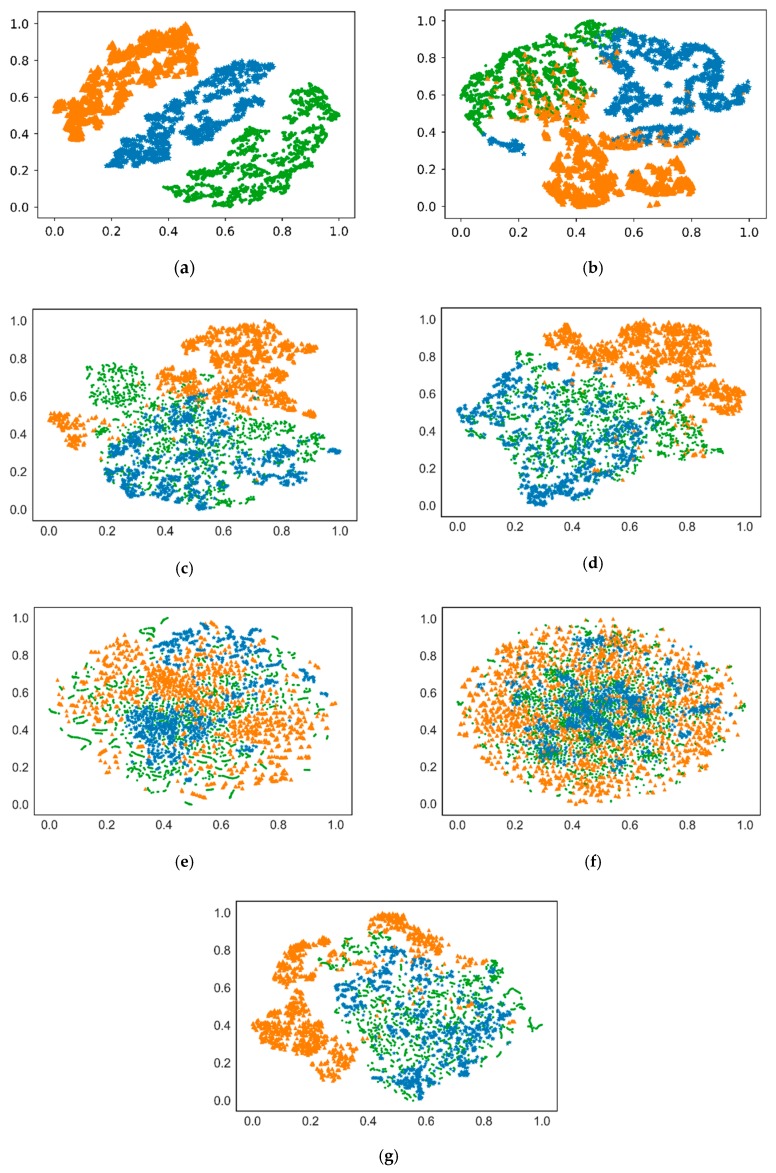
Dimension reduction distribution of training data ($H_{\mathrm{Sat}}>1000\mathrm{km},\mathrm{Cluster}=\mathrm{No}.3$) for these seven methods. (**a**) GRU neural network; (**b**) CNN; (**c**) Autoencoder (AE); (**d**) Denoising autoencoder (DAE); (**e**) PCA; (**f**) Dictionary learning (DL); (**g**) Manifold learning (ML).

Although the classification results of LSVM could prove the feature extraction validity of GRU neural network in some aspects, it is still expected to further display the distribution of extracted features for these seven recognition methods. Therefore, dimension reduction visualization technology is employed in this paper, which can map high dimensional feature data to two or three dimensions. At this time, the distribution of extracted features can be seen intuitively. Figure 13 shows dimension reduction distribution of features extracted based on one cluster training data ($H_{\mathrm{Sat}}>1000\mathrm{km},\mathrm{Cluster}=\mathrm{No}.3$) for these seven methods. It can be found in Figure 13a that the GRU neural network has the best feature distribution result because the dimension reduction features of three satellites are separated from each other. However, other methods have more or less intersections between different satellite classes, especially the last five methods. It is confirmed that the features extracted by the GRU neural network are more effective and highly discriminative.

## 5. Conclusions

In this paper, a novel satellite target recognition method based on radar data is proposed. It mainly includes two modules of data partition and a deep classification model. Satellite orbital altitude is introduced as a distinct and accessible feature because it is easy to calculate based on radar data and has relative stability. A hierarchical clustering method based on a new NADDCC distance metric is utilized to segment the radar observation angular domain. These two technologies can effectively complete data partition. Then, a GRU-SVM model is designed for radar HRRP satellite recognition, which is comprised of a deep GRU neural network as a feature extractor and LSVM as a classifier. The GRU neural network training records and feature visualization results all confirm that this GRU neural network could extract more deep and abstract features and these features have better separability. Furthermore, performance testing and comparison experiments also demonstrate that GRU neural network possesses better comprehensive performance for HRRP target recognition than LSTM neural network and conventional RNN; data partition can improve the recognition rate of satellites, and the recognition performance of our satellite target recognition method is almost better than that of other several common feature extraction methods or no data partition.

## Figures and Tables

**Table 1:** List of the acronyms used in this paper.

Acronym	Definition
GRU	Gated Recurrent Unit
HRRP	High Resolution Range Profile
SVM (LSVM)	(Linear) Support Vector Machine
RATR	Radar Automatic Target Recognition
ISAR	Inverse Synthetic Aperture Radar
NADDCC	Normalized Angular Distance Divided by Correlation Coefficient
PCA	Principal Component Analysis
CNN	Convolutional Neural Network
RNN	Recurrent Neural Network
LSTM	Long Short-Term Memory
MTRC	Migration Throuth Resolution Cells
RCS	Radar Cross Section
MAE	Mean Absolute Error
AE	AutoEncoder
DAE	Denoising AutoEncoder

**Table 2:** Parameters of the radar and satellites.

**Radar Parameters**	**Center Frequency**		10 GHZ
	**Bandwidth**		1 GHZ
	**Longitude**		115°
	**Latitude**		30.5°
	**Altitude**		0
**Satellite**	**Apogee Range (km)**	**Eccentricity**	**Inclination (°)**
No.1	390~425	$8{.1\;\times \;10}^{-4}$	51.6
No.2	300~320	$1{.0\;\times \;10}^{-7}$	51.0
No.3	310~322	$1{.1\;\times \;10}^{-3}$	54.5
No.4	721~730	$5{.0\;\times \;10}^{-3}$	57.0
No.5	630~643	$2{.1\;\times \;10}^{-4}$	97.8
No.6	615~624	$2{.2\;\times \;10}^{-4}$	97.9
No.7	670~682	$1{.1\;\times \;10}^{-3}$	98.1
No.8	1045~1165	$1{.3\;\times \;10}^{-3}$	63.4
No.9	1094~1112	$3{.5\;\times \;10}^{-3}$	123.0
No.10	1347~1355	$3{.2\;\times \;10}^{-4}$	58.0

**Table 3:** Recognition accuracy of this recognition method.

Orbital Altitude (km)	Cluster	Recognition Rate of Satellites (%)										Total Accuracy (%)
		No.1	No.2	No.3	No.4	No.5	No.6	No.7	No.8	No.9	No.10	
(0, 500]	No.1	99.7	98.9	99.4	/ ^1^	/	/	/	/	/	/	99.2
	No.2	99.7	99.6	99.5	/	/	/	/	/	/	/	
	No.3	97.0	90.5	95.7	/	/	/	/	/	/	/	
(500,1000]	No.1	/	/	/	99.0	99.7	99.8	99.2	/	/	/	
	No.2	/	/	/	99.4	99.7	99.7	98.8	/	/	/	
	No.3	/	/	/	99.6	99.4	99.8	99.2	/	/	/	
	No.4	/	/	/	100	99.6	99.7	98.9	/	/	/	
(1000,∞)	No.1	/	/	/	/	/	/	/	99.9	99.9	99.9	
	No.2	/	/	/	/	/	/	/	98.5	99.9	98.9	
	No.3	/	/	/	/	/	/	/	100	100	100	

^1^ There is no such satellite in the corresponding cluster.

**Table 4:** Performance comparison of three RNN-based neural network.

**Method**	**Orbital Altitude (km)**	**Cluster**	**Recognition Rate of Satellites (%)**										**Total Accuracy (%)**
			**No.1**	**No.2**	**No.3**	**No.4**	**No.5**	**No.6**	**No.7**	**No.8**	**No.9**	**No.10**	
GRU-SVM	(0, 500]	No.2	99.7	98.9	99.4	/ ^1^	/	/	/	/	/	/	99.6
RNN-SVM			70.2	43.5	86.8	/	/	/	/	/	/	/	66.9
LSTM-SVM			96.7	93.7	97.7	/	/	/	/	/	/	/	96.0
GRU-SVM	(500,1000]	No.1	/	/	/	99.0	99.7	99.8	99.2	/	/	/	97.8
RNN-SVM			/	/	/	99.1	82.9	87.5	80.7	/	/	/	86.9
LSTM-SVM			/	/	/	99.4	86.3	88.2	79.7	/	/	/	87.7
GRU-SVM	(1000,∞)	No.3	/	/	/	/	/	/	/	100	100	100	100
RNN-SVM			/	/	/	/	/	/	/	97.6	93.0	90.0	93.3
LSTM-SVM			/	/	/	/	/	/	/	96.1	99.4	93.1	96.0
GRU-SVM	(1000,∞)	× ^2^	/	/	/	/	/	/	/	99.0	99.9	98.7	99.2
RNN-SVM			/	/	/	/	/	/	/	86.7	93.9	55.3	78.7
LSTM-SVM			/	/	/	/	/	/	/	97.4	99.8	96.6	97.9
GRU-SVM	×	No.2	99.6	97.4	92.0	98.1	94.6	95.0	89.0	97.8	99.6	97.1	95.8
RNN-SVM			88.1	73.6	78.6	88.2	81.0	77.5	61.4	89.6	85.7	78.2	79.5
LSTM-SVM			95.9	93.9	83.6	90.4	86.9	80.9	69.3	87.5	98.1	87.9	87.0
**Method**	**Orbital Altitude (km)**	**Cluster**	**Training Data Scale**			**Epoch**		**CPU**		**GPU**		**Training Time**	
GRU	(0, 500]	No.2	55,440 HRRPs			100		E5-2630 32 G		NVIDIA Quadro P4000		21 h 36 min 16 s	
RNN												20 h 29 min 36 s	
LSTM												43 h 59 min 28 s	
GRU	(500,1000]	No.1	62,720 HRRPs			100		E5-2680 256 G		GTX 1080 Ti		26 h 32 min 11 s	
RNN												16 h 01 min 13 s	
LSTM												32 h 52 min 36 s	
GRU	(1000,∞)	No.3	36,400 HRRPs			100		E5-2630 32 G		NVIDIA Quadro P4000		33 h 11 min 04 s	
RNN												19 h 43 min 53 s	
LSTM												30 h 51 min 39 s	
GRU	(1000,∞)	×	84,000 HRRPs			100		E5-2680 256 G		GTX 1080 Ti		30 h 34 min 00 s	
RNN												17 h 17 min 48 s	
LSTM												36 h 08 min 35 s	
GRU	×	No.2	86,800 HRRPs			100		E5-2680 256 G		GTX 1080 Ti		37 h 43 min 18 s	
RNN												22 h 08 min 34 s	
LSTM												42 h 37 min 34 s	

^1^ There is no such satellite in the corresponding cluster. ^2^ This method or technology is not applied.

**Table 5:** The recognition accuracy of seven methods under different conditions.

Method	Orbital Altitude (km)	Cluster	Recognition Rate of Satellite (%)										Total Accuracy (%)
			No.1	No.2	No.3	No.4	No.5	No.6	No.7	No.8	No.9	No.10	
GRU-SVM	√ ^1^	√	99.1	97.4	99.7	99.7	99.6	99.8	99.1	99.1	99.9	99.6	99.2
	√	× ^2^	93.3	82.3	94.5	99.7	96.4	98.6	94.4	99.0	99.9	97.3	95.4
	×	√	97.2	82.8	89.4	97.7	89.3	92.4	84.8	93.8	99.5	90.7	91.7
	×	×	63.6	41.0	73.3	85.1	70.8	89.0	56.8	57.4	90.8	48.9	67.6
CNN-SVM	√	√	98.9	93.8	97.4	99.6	95.0	95.4	88.4	99.7	99.1	97.4	96.6
	√	×	97.3	88.1	96.6	98.8	90.4	93.7	81.2	93.3	99.2	88.9	93.1
	×	√	97.9	83.6	83.3	98.5	85.9	92.7	78.5	94.3	98.7	91.8	90.4
	×	×	76.9	82.6	82.0	78.6	70.1	70.6	70.8	77.6	63.0	59.1	72.6
AE-SVM	√	√	86.4	83.9	77.1	86.3	85.8	75.9	70.2	81.5	98.3	88.4	83.3
	√	×	81.0	85.0	79.0	76.0	83.0	70.0	64.0	72.0	93.0	82.0	77.9
	×	√	77.2	67.7	52.3	76.7	63.8	58.1	57.6	53.1	85.4	54.6	63.9
	×	×	69.0	57.0	43.0	64.0	49.0	43.0	40.0	39.0	78.0	43.0	52.0
DAE-SVM	√	√	85.3	80.4	73.0	88.1	75.1	76.7	69.8	78.5	95.6	79.0	80.0
	√	×	81.0	74.0	72.0	77.0	70.0	69.0	62.0	71.0	92.0	69.0	73.6
	×	√	73.6	58.6	45.4	77.8	51.8	56.3	53.0	50.8	76.5	53.3	60.3
	×	×	64.0	46.0	39.0	66.0	39.0	47.0	40.0	38.0	70.0	42.0	49.0
PCA-SVM	√	√	89.4	84.5	77.7	90.2	85.9	79.8	77.7	81.9	97.5	91.0	86.7
	√	×	83.0	77.0	77.0	76.0	82.0	70.0	66.0	74.0	93.0	82.0	77.6
	×	√	79.1	64.6	52.1	78.0	64.5	54.9	55.4	50.8	75.7	66.5	66.0
	×	×	71.0	48.0	38.0	66.0	51.0	44.0	46.0	38.0	82.0	51.0	54.0
DL-SVM	√	√	87.4	80.0	75.2	89.4	76.4	76.0	74.4	78.5	96.9	85.0	81.2
	√	×	82.0	75.0	70.0	77.0	72.0	67.0	60.0	72.0	92.0	75.0	74.9
	×	√	74.4	53.8	47.3	77.3	55.7	53.1	56.5	51.9	77.2	57.4	60.6
	×	×	65.0	39.0	34.0	65.0	45.0	42.0	42.0	37.0	73.0	42.0	49.0
ML-SVM	√	√	87.6	83.4	79.1	92.6	80.1	80.2	75.2	79.7	97.0	81.3	83.6
	√	×	83.0	78.0	74.0	82.0	74.0	73.0	67.0	72.0	93.0	73.0	77.3
	×	√	74.3	62.7	54.0	82.7	56.3	57.3	56.3	52.8	78.1	59.2	63.3
	×	×	68.0	47.0	43.0	74.0	44.0	47.0	44.0	38.0	73.0	46.0	53.0

^1^ This method or technology is applied. ^2^ This method or technology is not applied.

## Appendix A. Satellite Orbital Altitude Calculation Method

Satellite orbital altitude could be obtained by multiple coordinate transformation based on radar measurement data. The Figure 4 shows these coordinate transformation diagrams. It mainly involves the following coordinate systems:

(1) Geocentric Cartesian coordinate system: its origin $O_{E}$ is situated at the center of the Earth. $O_{E}X_{E}$ axis is located in the equatorial plane and points to the meridian at the Greenwich Observatory. $O_{E}Z_{E}$ axis is perpendicular to the equatorial plane, coincident with Earth’s rotation axis and points to the north pole. $O_{E}Y_{E}$ axis is located in the equatorial plane and its direction satisfies the right-handed rectangular coordinate system criterion.
(2) Radar body Cartesian coordinate system: its origin S is situated at the radar station. ${\mathrm{SX}}_{S}$ axis is located in the ground plane of the radar station and points to the south. ${\mathrm{SY}}_{S}$ axis is located in the ground plane of the radar station and points to the east. ${\mathrm{SZ}}_{S}$ axis passes the radar station, along its plumb line and points to the zenith.

The detailed coordinate transformation process is as follows:

(a) Conversion of radar polar coordinate system into radar body Cartesian coordinate system

Set the coordinates of satellite target in the radar polar coordinate system to (ρ, θ, ε), where ρ is the slant distance from satellite to radar station. θ, ε represent the radar observation azimuth and elevation angle, respectively. These parameters could be obtained in the process of radar measurement. Then, the coordinates $\left(x_{1}{,\;y}_{1}{,\;z}_{1}\right)$ of the satellite in the radar body Cartesian coordinate system could be calculated by the following formula:

(A1) $$\{\begin{array}{l} x_{1}\;=\;\rho \cdot \cos\theta \cdot \cos\varepsilon \\ y_{1}\;=\;\rho \cdot \sin\theta \cdot \cos\varepsilon \\ z_{1}\;=\;\rho \cdot \sin\varepsilon \end{array}$$

(b) Conversion of geodetic coordinate system to geocentric Cartesian coordinate system

Set the geodetic coordinates of the radar station are $\left(\lambda_{S}{,\;\varphi }_{S},\;H\right)$, where $\lambda_{S}$, $\varphi_{S}$, H represent the longitude, latitude and altitude of the radar station, respectively, which are known for certain radar station. Its corresponding coordinates $\left(x^{\prime }{,\;y}^{\prime }{,\;z}^{\prime }\right)$ in the geocentric Cartesian coordinate system can be computed by the following formula:

(A2) $$\{\begin{array}{c} x^{\prime }\;=\;\left(N+H\right)\cdot {\cos\lambda }_{S}\cdot {\cos\varphi }_{S}\\ y^{\prime }\;=\;\left(N+H\right)\cdot {\sin\lambda }_{S}\cdot {\cos\varphi }_{S}\\ z^{\prime }\;=\;\left(N\cdot \left({1-e}^{2}\right)+H\right)\cdot {\sin\varphi }_{S} \end{array}$$

where N denotes the radius of prime vertical and is calculated by $N=a/\left({1-e}^{2}\sin^{2}\varphi_{S}\right)$. a, e represent the semi-major axis and oblateness of earth.

(c) Conversion of radar body Cartesian coordinate system to geocentric Cartesian coordinate system

Set the coordinates of satellite in the radar body Cartesian coordinates system are $\left(x_{1}{,\;y}_{1}{,\;z}_{1}\right)$, suppose its corresponding coordinates in the geocentric Cartesian coordinates system are $\left(x_{e}{,\;y}_{e}{,\;z}_{e}\right)$, then the conversion formula is as:

(A3) $$\left[\begin{array}{c} x_{e}\\ y_{e}\\ z_{e} \end{array}\right]{\;=\;R}_{T}\left[\begin{array}{c} x_{1}\\ y_{1}\\ z_{1} \end{array}\right]\;+\;\left[\begin{array}{c} x\prime \\ y\prime \\ z\prime \end{array}\right]$$

where $R_{T}$ is rotation matrix and calculated as:

(A4) $$R_{T}\;=\;\left[\begin{array}{ccc} {\sin\varphi }_{S}{\cos\lambda }_{S} & {-\sin\lambda }_{S} & {\cos\varphi }_{S}{\cos\lambda }_{S}\\ {\sin\varphi }_{S}{\sin\lambda }_{S} & {\cos\lambda }_{S} & {\cos\varphi }_{S}{\sin\lambda }_{S}\\ {-\cos\varphi }_{S} & 0 & {\sin\varphi }_{S} \end{array}\right]$$

Once the coordinates of satellite in the geocentric Cartesian coordinate system are known, it is easy to calculate the altitude of the satellite. The calculation formula is as follows:

(A5) $$H_{\mathrm{sat}}\;=\;\sqrt{{\left(x_{e}\right)}^{2}+{\left(y_{e}\right)}^{2}+{\left(z_{e}\right)}^{2}}{\;-\;R}_{e}$$
